# Multi-Oriented and Scale-Invariant License Plate Detection Based on Convolutional Neural Networks

**DOI:** 10.3390/s19051175

**Published:** 2019-03-07

**Authors:** Jing Han, Jian Yao, Jiao Zhao, Jingmin Tu, Yahui Liu

**Affiliations:** 1 School of Remote Sensing and Information Engineering, Wuhan University, Wuhan 430070, China; j.han@whu.edu.cn (J.H.); jiao.zhao@whu.edu.cn (J.Z.); jingmin.tu@whu.edu.cn (J.T.); yahui.liu@unitn.it (Y.L.); 2 School of Sociology, Wuhan University, Wuhan 430070, China

**Keywords:** convolutional neural networks, deep learning, license plate detection, multi-orientation, multi-scale detection

## Abstract

License plate detection (LPD) is the first and key step in license plate recognition. State-of-the-art object-detection algorithms based on deep learning provide a promising form of LPD. However, there still exist two main challenges. First, existing methods often enclose objects with horizontal rectangles. However, horizontal rectangles are not always suitable since license plates in images are multi-oriented, reflected by rotation and perspective distortion. Second, the scale of license plates often varies, leading to the difficulty of multi-scale detection. To address the aforementioned problems, we propose a novel method of multi-oriented and scale-invariant license plate detection (MOSI-LPD) based on convolutional neural networks. Our MOSI-LPD tightly encloses the multi-oriented license plates with bounding parallelograms, regardless of the license plate scales. To obtain bounding parallelograms, we first parameterize the edge points of license plates by relative positions. Next, we design mapping functions between oriented regions and horizontal proposals. Then, we enforce the symmetry constraints in the loss function and train the model with a multi-task loss. Finally, we map region proposals to three edge points of a nearby license plate, and infer the fourth point to form bounding parallelograms. To achieve scale invariance, we first design anchor boxes based on inherent shapes of license plates. Next, we search different layers to generate region proposals with multiple scales. Finally, we up-sample the last layer and combine proposal features extracted from different layers to recognize true license plates. Experimental results have demonstrated that the proposed method outperforms existing approaches in terms of detecting license plates with different orientations and multiple scales.

## 1. Introduction

License plate recognition is a key technology for intelligent transportation systems. It has been widely applied in traffic surveillance and road management. Typically, license plate recognition consists of three procedures: license plate detection (LPD), character segmentation, and text recognition. Among them, LPD is the first and key step, since it directly affects follow-up tasks and determines the overall accuracy [1]. As the need for automatic detection of motor vehicles grows, to design an effective and efficient LPD method is becoming increasingly important.

To detect license plates in images, representative features should be extracted to distinguish the target license plates from the background. Traditional LPD methods carefully handcraft features based on *inherent attributes* of license plates (e.g., texture [2,3,4,5], boundary [6,7,8], color [9,10,11] and character [12,13,14,15]) or *general feature descriptors* (e.g., scale-invariant feature transform (SIFT) [16], speeded-up robust features (SURF) [17], histogram of oriented gradient (HOG) and its various variants [18,19,20]). These methods can achieve satisfactory performance under certain conditions. However, the handcrafting process is labor-intensive, and the extracted features only reveal local and low-level characteristics [21].

In the practical use, multiple kinds of features are often combined to improve the accuracy. For example, Yuan et al. [22] proposed an algorithm that hybridized boundary and color features. They first down-scaled the input images and applied a line density filter to extract candidate regions. Then they trained a cascaded classifier with color saliency features and used the classifier to detect true license plates. This model is accurate and achieves state-of-the-art performance among traditional LPD methods. Therefore, it is chosen as representative of the traditional LPD methods to be compared with our approach in the experiments.

In recent years, deep learning methods based on convolutional neural networks (CNN) have achieved remarkable performance in general object-detection tasks. Driven by the great success, several methods [23,24,25,26] have been proposed to adapt the object-detection algorithms for the LPD task. In CNN, features are automatically learned from data and reveal high-level characteristics of the inputs. Therefore, LPD methods based on deep learning are less labor-intensive and more accurate than traditional ones. In [23], character regions were first recognized by a text/non-text CNN classifier, and license plate regions were further located by a plate/non-plate CNN classifier. To improve the efficiency, Rafique et al. [26] used the more advanced structure of Faster R-CNN [27] which directly detected the license plates in an end-to-end manner. Faster R-CNN [27] is the classic algorithm among region-based deep learning methods [27,28,29,30]. It consists of a region proposal network (RPN) to generate high-quality proposals, and a detection network to recognize and locate true objects. Other mainstream object-detection methods are proposal-free algorithms such as YOLO [31] and SSD [32]. These proposal-free methods directly estimate object locations without generating region proposals. Therefore, they are faster but relatively less accurate and robust than the region-based ones. Our work adopts the region-based mechanism and uses Faster R-CNN [27] as the backbone architecture.

However, directly training the deep models for the LPD task may not achieve good enough performance. For the practical LPD applications, there still exist two main challenges.

**Figure 1:**
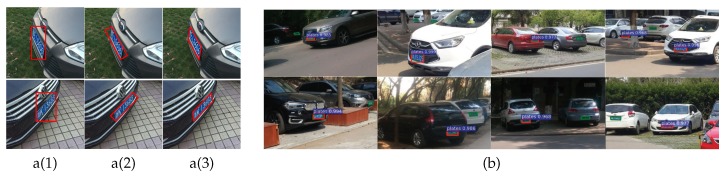
Two main challenges for existing methods. (**a**) Comparison between different bounding methods (horizontal rectangles, rotated rectangles, and our bounding parallelograms). Existing methods based on horizontal or rotated rectangles cannot tightly enclose the multi-oriented license plates. (**b**) Representative detection results of Faster R-CNN [27] trained for LPD. The detected regions and actual license plates are presented by red horizontal rectangles and green polygons, respectively. It is difficult to detect license plates with multiple scales, especially the tiny ones.

The first challenge is that the detected regions are not accurate enough. Unlike general object-detection, LPD is often the prerequisite for character recognition. Since the license plates should be rectified ahead of recognition, the localization needs to be highly accurate. However, general object-detection algorithms enclose objects by horizontal rectangles. As shown in Figure 1a(1), horizontal rectangles cannot tightly enclose the multi-oriented license plates.

Works for text detection [33,34,35,36,37] can provide some insights for the multi-oriented detection issue. In [33,34], fully convolutional network (FCN) [38] was used to predict salient maps, and geometric approaches were applied to estimate the orientations. These methods partly solve the problem, but the prerequisite segmentation is time-consuming. To improve the efficiency, end-to-end systems based on detection networks have been proposed in [35,36]. These methods adapted the object-detection networks to directly regress rotated rectangles from inclined proposals or boxes. They are relatively more efficient than the segmentation-based methods, but the added inclined hypotheses still produce heavy computation cost. More importantly, the rotated rectangles are still not accurate enough for practical applications. As shown in Figure 1a(2), rotated rectangles have right angles, while the skewed license plates have free angles. The mismatch results in inaccurate enclosure around the corners. Recently, Liao et al. [37] proposed a method that enclosed text regions with the highly accurate quadrilaterals. Since quadrilaterals have free orientations and angles, they can fit arbitrary regions. However, the lack of geometric constraints brings difficulty to model training and harms the recognition performance. Besides, its proposal-free architecture is less accurate and robust than the region-based ones. Therefore, ref. [37] fails to guarantee the localization precision and detection accuracy simultaneously.

The second challenge is that the detection of license plates with multiple scales has not been well solved. A traditional way to improve the scale invariance is to use the scattering operator as in the works of [39,40,41]. The scattering descriptor contains high-frequency information of the wavelet coefficients, and is robust to the scale variance of inputs. In deep learning methods, Faster R-CNN [27] deals with the scale issue simply by referring to anchor boxes with multiple scales and aspect ratios. This is effective in some way, but there is still a severe inconsistency between the objects with various scales, and filter receptive fields with very limited scale ranges [42]. As shown in Figure 1b, the detection performance is particularly poor for small targets. To improve the performance, He et al. [43] built image pyramids with multiple scales. This method works well, but it requires inputs with multiple scales, which brings high computational cost. To improve the efficiency, works of [32,42,44,45] built feature pyramids instead of image pyramids by exploiting different convolutional layers. In [32,42], region proposals or default boxes were generated on multiple layers. In [44,45,46], features were extracted from different layers. These methods have achieved good performance and are inspiring to our work. However, they only take advantage of the multiple layers for one task, while the layers can be further used.

To address the above-mentioned two problems, we propose a novel method of multi-oriented and scale-invariant license plate detection (MOSI-LPD) based on CNNs. The proposed MOSI-LPD tightly encloses license plates with bounding parallelograms, and is highly invariant to the scale discrepancy of license plates. In addition, the proposed MOSI-LPD is robust to challenging conditions with a comparable detection speed. The main contributions of our work can be summarized as follows.

- We propose novel strategies to tightly enclose the multi-oriented license plates with bounding parallelograms. Both the network architecture and the loss function are elaborately designed to directly regress bounding parallelograms from horizontal proposals. Our method significantly improves the localization precision and guarantees a high detection accuracy simultaneously.
- We design effective strategies to detect license plates with multiple scales. Multiple convolutional layers are exploited both for proposal generation and feature extraction. The priori knowledge regarding inherent shapes of license plates is considered for anchor box design. Our method is highly invariant to the scale discrepancy of license plates, and effectively detects tiny license plates that are only several pixels.
- We construct a large license plate dataset. The dataset contains more than 7000 images, and all the license plates are labeled by the exact edge points. The dataset is publicly available for related research (http://cvrs.whu.edu.cn/projects/LASI-LPL/).

## 2. Materials and Methods

### 2.1. Overall Structure

**Figure 2:**
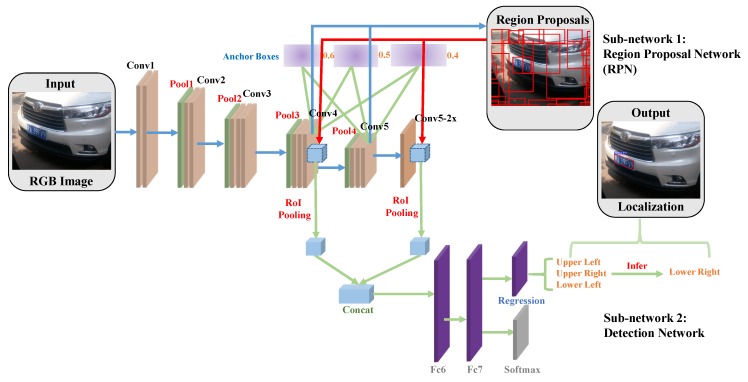
The overall structure of our MOSI-LPD. The backbone framework is Faster R-CNN [27], the classic region-based deep learning network for object detection. To achieve multi-oriented and scale-invariant detection, several vital modifications are proposed. For the RPN sub-network, license plate proposals are generated on both the “Conv5” layer and the “Conv4” layer to combine and produce stronger proposals. The anchor boxes are set based on the priori knowledge regarding inherent shapes of license plates. For the detection sub-network, RoI pooling is conducted on the combined layers of the up-sampled “ Conv5-2x” layer and “Conv4” layer. We estimate three edge points of the license plates by regressing relative positions from horizontal proposals. The fourth edge point is inferred based on the symmetry property to form final bounding parallelograms that tightly enclose the multi-oriented license plates.

The overall structure of our MOSI-LPD is illustrated in Figure 2. Following the basic architecture of Faster R-CNN [27], our MOSI-LPD consists of two sub-networks: (i) a RPN generating proposals that probably contain a license plate; (ii) a detection network recognizing positive proposals and regressing accurate locations. The two sub-networks share the fundamental CNN structure.

In details, the fundamental shared CNN structure has five convolutional layers, each of which is followed by a Rectified Linear Unit (ReLU) layer. Right after the first four convolutional layers, there is also a Max-pooling layer. To construct RPN, we slide a 3×3 filter over the “Conv4” and “Conv5” layers, respectively. In each sliding window, the 512-dimension convolutional features are extracted, and we simultaneously refer to 9 anchor boxes. The scales and aspect ratios of the anchor boxes are set based on the priori knowledge regarding the inherent shapes of license plates. The features are then fed into two fully connected layers: the first layer classifies the regions as license plate proposals or the background, and the second layer regresses functions that adjust proposal positions.

The combined license plate proposals raised by RPN act as the inputs of the detection sub-network. They are projected onto the combined layers of the up-sampled “Conv5-2x” layer and “Conv4” layer. Similarly, the fused features are fed into two fully connected layers. The first layer classifies the proposals as license plates or the background, and the second layer regresses functions that map the proposals to license plates positions. Final outputs of our MOSI-LPD are bounding parallelograms that tightly enclose the multi-oriented license plates.

Our strategies to achieve multi-oriented and scale-invariant detection are detailed as follows.

### 2.2. Multi-Oriented Detection Based on Bounding Parallelograms

Existing methods enclose license plates with horizontal rectangles. However, for practical LPD applications, the license plates in images are multi-oriented reflected by rotation and perspective distortions. As illustrated in Figure 1a(1), the horizontal-oriented rectangles cannot tightly enclose the multi-oriented license plates. An inspiring solution may be predicting rotated rectangles as done in several text detection methods [35,36]. However, Figure 1a(2) shows that rotated rectangles still fail to enclose the corners of the license plates well. This is attributed to the inconsistency between the fixed angles of the rotated rectangles and the varying angles of the license plates. Hence, it is obvious that both horizontal and rotated rectangles are not suitable for practical LPD applications, and it is of significant importance to develop more precise bounding techniques. In this paper, we propose a new method for the LPD task by tightly enclosing license plates with bounding parallelograms (shown in Figure 1a(3)).

To achieve this goal, we first reformat edge point coordinates of the license plates. For each license plate, we reformulate the coordinates of the upper left point $\left(x_{U}^{l},y_{U}^{l}\right)$, upper right point $\left(x_{U}^{r},y_{U}^{r}\right)$ and lower left point $\left(x_{L}^{l},y_{L}^{l}\right)$ into $\left(x+w_{1},y+h_{1}\right)$, $\left(x+w_{2},y+h_{2}\right)$ and $\left(x+w_{3},y+h_{3}\right)$, respectively, where x,y are the coordinates of the parallelogram central point, and $w_{1},h_{1},w_{2},h_{2},w_{3},h_{3}$ are the relative positions of $\left(x_{U}^{l},y_{U}^{l}\right)$, $\left(x_{U}^{r},y_{U}^{r}\right)$, $\left(x_{L}^{l},y_{L}^{l}\right)$ to the central point. Considering the symmetry property, the last lower right point $\left(x_{L}^{r},y_{L}^{r}\right)$ can be inferred as follows:

(1) $$\left\{\begin{array}{cc} x_{L}^{r} & =x_{U}^{r}+x_{L}^{l}-x_{U}^{l}\\ y_{L}^{r} & =y_{U}^{r}+y_{L}^{l}-y_{U}^{l}. \end{array}\right.$$

Please note that there are three key points in the reformulation strategy. (i) The first is that we enclose license plates with parallelograms rather than quadrilaterals to simplify the task and help model training. Although quadrilaterals can indicate precise locations, their structures are over-free without any geometric constraints. To form quadrilaterals, the network must learn all the edge points and the lack of geometric constraints brings great difficulty to model training. By contrast, to form parallelograms, the lower right point $\left(x_{L}^{r},y_{L}^{r}\right)$ can be easily estimated via Equation (1) based on the symmetry property. Besides, the geometric constraints are enforced in the loss function to help model training. In fact, experimental results in Section 3 have shown that “bounding parallelograms” are precise enough: detection results of our MOSI-LPD achieve an average overlap of over 0.85 with actual license plates, outperforming the “horizontal rectangles” by more than 15%. (ii) The second is that we regress three edge points rather than two. Although two edge points and a center point are enough to determine a parallelogram, we employ three edge points to leverage the symmetry constraints. Since the upper right point $\left(x_{U}^{r},y_{U}^{r}\right)$ and lower left point $\left(x_{L}^{l},y_{L}^{l}\right)$ are symmetric around the central point, $w_{2}$ and $w_{3}$ should add up to zero, and the same goes for $h_{2}$ and $h_{3}$. The constraints are imposed in the loss function to facilitate model training. (iii) The last is that we parameterize edge points by their relative positions to the central point. Absolute coordinates are not used because they contain no relative information and are very difficult to learn in practice. All these techniques are crucial for network training, since the model cannot converge if we leave out any of them.

To regress the license plate location parameterized by central point coordinates and relative positions $\left(p_{x}^{*},p_{y}^{*},p_{w_{1}}^{*},p_{h_{1}}^{*},p_{w_{2}}^{*},p_{h_{2}}^{*},p_{w_{3}}^{*},p_{h_{3}}^{*}\right)$ from a proposal parameterized by central point coordinates and side lengths $\left(p_{x},p_{y},p_{w},p_{h}\right)$, the mapping functions should be learned. Inspired by [28], we formulate the mapping functions to: $d_{x}\;=\;\frac{p_{x}^{*}-p_{x}}{p_{w}},d_{y}\;=\;\frac{p_{y}^{*}-p_{y}}{p_{h}},d_{w_{1}}\;=\;\log\left(\frac{p_{w_{1}}^{*}}{p_{w}}\right),d_{h_{1}}\;=\;\log\left(\frac{p_{h_{1}}^{*}}{p_{h}}\right),d_{w_{2}}\;=\;\log\left(\frac{p_{w_{2}}^{*}}{p_{w}}\right),d_{h_{2}}\;=\;\log\left(\frac{p_{h_{2}}^{*}}{p_{h}}\right),d_{w_{3}}\;=\;\log\left(\frac{p_{w_{3}}^{*}}{p_{w}}\right),d_{h_{3}}\;=\;\log\left(\frac{p_{h_{3}}^{*}}{p_{h}}\right)$, where $d_{x}$ and $d_{y}$ specify scale-invariant translations between the central points, and $d_{w_{1}},d_{h_{1}},d_{w_{2}},d_{h_{2}},d_{w_{3}},d_{h_{3}}$ specify the log-space translations between the relative positions and the side lengths.

To learn the above-mentioned parameters denoted as *W*, we train the model on training batches $S={\left\{\left(X_{i},Y_{i}\right)\right\}}_{i=1}^{N}$ consisting of *N* samples, where $X_{i}$ represents each image patch and $Y_{i}$ is the combination of its class label $y_{i}$ (“1” for license plates and “0” for background) and regression targets ${\hat{d}}_{i}$. Based on stochastic gradient descent (SGD), we minimize a joint multi-task loss function defined as:

(2) $$\begin{array}{cc} L(W)\;\;=\;\; & \lambda_{1}\sum_{i=1}^{N}L_{\mathrm{cls}}\left(p_{i},y_{i}\right)+\lambda_{2}\sum_{i=1}^{N}\left[y_{i}\;=\;1\right]L_{\mathrm{reg}}\left(d_{i},{\hat{d}}_{i}\right)\\ & +\lambda_{3}\sum_{i=1}^{N}\left[y_{i}\;=\;1\right]L_{\mathrm{sym}}\left(w_{i}^{2},w_{i}^{3}\right)+\lambda_{4}\sum_{i=1}^{N}\left[y_{i}\;=\;1\right]L_{\mathrm{sym}}\left(h_{i}^{2},h_{i}^{3}\right), \end{array}$$

where $L_{\mathrm{cls}}$ is the classification loss, $p_{i}=\left(p_{0},p_{1}\right)$ is the probability over the background and license plate class provided by the classifiers, $L_{\mathrm{reg}}$ is the regression loss, $d_{i}$ and ${\hat{d}}_{i}$ are the predicted and ground-truth regression targets, respectively, $L_{\mathrm{sym}}$ are our symmetry constraints, $w_{i}^{2},h_{i}^{2}$ are the relative positions of the upper right point, $w_{i}^{3},h_{i}^{3}$ are the relative positions of the lower left point, and $\lambda_{1},\lambda_{2},\lambda_{3},\lambda_{4}$ are the trade-off coefficients. Since negative samples will turn the term of $\left[y_{i}=1\right]$ to 0, its regression loss and symmetry constraints are dropped, which makes sense that there is no ground-truth parallelogram for the background class. More detailed designs of the loss function for the two sub-networks are introduced in the following.

For the RPN sub-network, we define the classification loss $L_{\mathrm{cls}}$ with the cross-entropy loss formulated as:

(3) $$L_{\mathrm{cls}}\left(p_{i},y_{i}\right)=-\log p_{y_{i}},$$

and the regression loss $L_{\mathrm{reg}}$ is defined as:

(4) $$L_{\mathrm{reg}}\left(d_{i},{\hat{d}}_{i}\right)=\frac{1}{4}\sum_{k\in \{x,y,w,h\}}{\mathrm{smooth}}_{L_{1}}\left(d_{i}^{k}-{\hat{d}}_{i}^{k}\right),$$

where $d_{i}=\left(d_{i}^{x},d_{i}^{y},d_{i}^{w},d_{i}^{h}\right)$ and ${\hat{d}}_{i}=\left({\hat{d}}_{i}^{x},{\hat{d}}_{i}^{y},{\hat{d}}_{i}^{w},{\hat{d}}_{i}^{h}\right)$ are the learned and ground-truth regression targets, respectively. In RPN, its regression targets are the transformations proposed in Fast R-CNN [30] to map an anchor box to a license plate proposal, and ${\mathrm{smooth}}_{L_{1}}$ is the robust loss function defined as:

(5) $${\mathrm{smooth}}_{L_{1}}\left(x\right)=\left\{\begin{array}{cc} 0.5x^{2} & \mathrm{if}\;|x|<1\\ |x|-0.5 & \mathrm{otherwise}. \end{array}\right.$$

Since region proposals do not contain relative positions of the edge points, the symmetry constraints are dropped by setting $\lambda_{3}$ and $\lambda_{4}$ to 0, and $\lambda_{1}$ and $\lambda_{2}$ are set to 1 to balance the classification and regression loss.

For the detection sub-network, the classification loss $L_{\mathrm{cls}}$ is defined in the same way as RPN, and we still calculate the regression loss with the ${\mathrm{smooth}}_{L_{1}}$ loss. However, as the regression targets have turned to involve eight mapping functions ${\hat{d}}_{i}=\left({\hat{d}}_{i}^{x},{\hat{d}}_{i}^{y},{\hat{d}}_{i}^{w_{1}},{\hat{d}}_{i}^{h_{1}},{\hat{d}}_{i}^{w_{2}},{\hat{d}}_{i}^{h_{2}},{\hat{d}}_{i}^{w_{3}},{\hat{d}}_{i}^{h_{3}}\right)$, which specify transformations from a license plate proposal to three edge points of a license plate as introduced above, the regression loss is formulated as:

(6) $$L_{\mathrm{reg}}\left(d_{i},{\hat{d}}_{i}\right)=\frac{1}{8}\sum_{k\in S}{\mathrm{smooth}}_{L_{1}}\left(d_{i}^{k}-{\hat{d}}_{i}^{k}\right).$$

where $S\;=\;(x,y,w_{1},h_{1},w_{2},h_{2},w_{3},h_{3})$, and $d_{i}=\left(d_{i}^{x},d_{i}^{y},d_{i}^{w_{1}},d_{i}^{h_{1}},d_{i}^{w_{2}},d_{i}^{h_{2}},d_{i}^{w_{3}},d_{i}^{h_{3}}\right)$ is the set of learned mapping functions.

To leverage the symmetry property of parallelograms, the geometric constraints are enforced through the symmetry loss $L_{\mathrm{sym}}$. Since the upper right point and lower left point of the license plates should be symmetric around the central point, $w_{i}^{2}$ and $w_{i}^{3}$ should add up to zero, and the same goes for $h_{i}^{2}$ and $h_{i}^{3}$. Therefore, we define the symmetry loss as:

(7) $$\left\{\begin{array}{c} L_{\mathrm{sym}}\left(w_{i}^{2},w_{i}^{3}\right)=w_{i}^{2}+w_{i}^{3}\\ L_{\mathrm{sym}}\left(h_{i}^{2},h_{i}^{3}\right)=h_{i}^{2}+h_{i}^{3}. \end{array}\right.$$

Given that the regressing task is more complex and challenging in the detection sub-network, we enhance effects of the regression loss and the symmetry constraints by setting the weight parameters $\lambda_{2},\lambda_{3},\lambda_{4}$ to 10 and assign 1 to $\lambda_{1}$. The imbalanced weights strengthen the learning of the regression process.

With the learned transformation functions, license plate proposals raised by RPN are mapped to three edge points of a nearby license plate parallelogram. The fourth edge point is inferred with (1) to form final bounding parallelograms that tightly enclose the multi-oriented license plates.

### 2.3. Scale-Invariant Detection

In practical LPD applications, license plates in images vary greatly in scale and it is difficult to achieve multi-scale detection. The original Faster R-CNN [27] deals with the scale issue simply by referring to anchor boxes with different scales and aspect ratios. The scale variance of the anchor boxes can help multi-scale detection to some extent, but there is still a severe inconsistency between the license plates with varying scales, and filter receptive fields with limited scale ranges. As shown in Figure 1b, Faster R-CNN [27] trained for LPD cannot effectively detect license plates with multiple scales.

For a more in-depth investigation, we can observe that the detection performance is particularly poor on small license plates. The reason is that the receptive fields become larger as the network goes deeper. For the tiny license plates, the re-projected area into the original image may contain high proportion of redundant information. The lack of necessary information seriously interferes with the detection process. To improve the scale invariance of the network, we introduce two strategies in the following.

Firstly, in the RPN sub-network, we generate proposals for candidate license plate regions on multiple output layers rather than only on the last convolutional layer. This is motivated by the fact that different convolutional layers have receptive fields with different resolutions. The lower layers have smaller receptive fields and extract local features. By contrast, the higher layers have bigger receptive fields and extract global features. Therefore, the lower layers are more suitable to detect small license plates, and the higher layers are more appropriate for detecting large license plates. In order to effectively detect license plates with multiple scales, we take advantage of both the lower “Conv4” layer and the higher “Conv5” layer when searching for region proposals. The results are combined to produce stronger proposals with complementary scales.

In addition, to generate proposals that better match the license plate shapes, we design the anchor boxes by considering the inherent shapes of license plates. Specifically, we analyze the scale ranges of the license plates, and set scales of the anchor boxes as 64×64, 128×128 and 256×256 based on the most common scales. We further analyze aspect ratios of the multi-oriented license plates. According to the statistical analysis, 0.4, 0.5 and 0.6 are chosen as the aspect ratios for the anchor boxes. Owing to the careful design based on prior knowledge, the generated proposals better fit the license plate regions.

Secondly, in the detection sub-network, different convolutional layers with various resolutions are also exploited to extract better features. We up-sample the “Conv5” layer to the size of the “Conv4” layer via the deconvolution operation, and combine the up-sampled layer “Conv5-2x” with the “Conv4” layer. For the feature extraction of the raised proposals, we perform RoI pooling on the combined layers, which shows better performance than traditional method that projects the proposals only to the last “Conv5” layer. We take this step by leveraging the fact that “Conv5” features are more appropriate for detecting license plates with normal-to-large scales, but less representative for small license plates. To obtain more detailed clues about license plates with small scales, we exploit the finer-grained “Conv4” features to get more local information.

## 3. Results

We have conducted extensive experiments to evaluate the proposed MOSI-LPD. In this section, we first introduce our dataset for training and testing. Next, we give implementation details and evaluation criteria. Finally, we compare and analyze performance between our MOSI-LPD and the baseline algorithms. The experimental results demonstrate that our MOSI-LPD tightly encloses the multi-oriented license plates with bounding parallelograms, and effectively detects license plates with multiple scales. Besides, our MOSI-LPD is highly robust to challenging conditions and achieves a comparable detection speed.

### 3.1. Dataset

**Figure 3:**
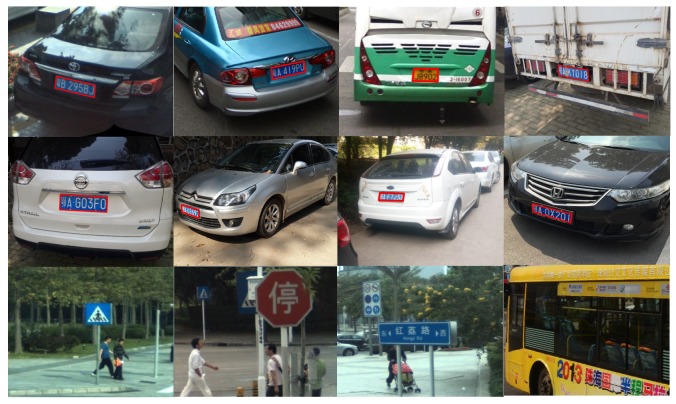
Sample images of our license plate dataset. First two rows: positive samples containing license plates with different orientations and multiple scales. All the license plates were manually labeled by the exact four edge points. Third row: negative samples containing objects similar to license plates.

To the best of our knowledge, existing license plate datasets annotate the license plates with horizontal rectangles. However, the information is not accurate enough to indicate actual positions of the multi-oriented license plates. Therefore, we constructed our own dataset. We manually collected 7284 images with totally 10,279 license plates, which had different orientations and multiple scales. We manually labeled the license plates with the exact four exact edge points. Since deep models should be trained with huge amounts of data, we further augmented the dataset by flipping, rotation (each image was rotated by 5, 10, 15, 20, 25, 30 degrees both in clockwise and counter clockwise orientations), blurring, brightening, color and contrast enhancement, and noise-adding. These processes extended the dataset to a total number of 131,112 images with 185,022 license plates. We also collected some negative samples containing objects similar to license plates (such as traffic signs, trademarks, and banners). Figure 3 shows some representative sample images of our dataset.

We divided the images into training set and testing set by randomly sampling 75% of them for training and using the rest for testing. For each experiment, we built test subsets according to its aim.

### 3.2. Implementation

The environment to implement our algorithm was a desktop running Ubuntu with TitanX. We initialized the fundamental building blocks with the VGG16 model pre-trained on ImageNet [47], and weights of the modified and added layers were initialized by the Xavier method [48]. The parameters were fine-tuned or learned from scratch via SGD. We followed the 4-step training scheme of Faster R-CNN [27] to train the net. Each of the four training stages contained 10k iterations, and the basic learning rate was set to 0.005 and decreased by 0.2 after each 3k iterations. The momentum and weight decay were configured as 0.9 and 0.0005, respectively.

### 3.3. Evaluation Criteria

Since there are no uniform criteria for LPD evaluation, we follow the text detection measurements and further include the intersection over union (IoU) metric to set comprehensive evaluation criteria for LPD.

The text detection measurements consist of **precision**, **recall** and **f-measure**, which are defined as:

(8) $$\left\{\begin{array}{c} Precision=\frac{TP}{TP+FP}\\ Recall=\frac{TP}{TP+FN}\\ F-measure=\frac{2\times Precision\times Recall}{Precision+Recall}, \end{array}\right.$$

where TP and FP are the numbers of correctly and falsely detected results, respectively, and FN is the number of missed targets. Generally, recall reflects the ability to detect the objects while precision indicates the probability of detection results to be true. It is highly desirable to obtain both high recall and high precision, but they are negatively correlated. For an overall evaluation, f-measure combines precision and recall by calculating the harmonic mean of them.

Since a major goal of our work is to enclose the multi-oriented license plates in a tighter way, we further take the IoU metric to measure the overlaps between the detected regions and the ground-truth license plate regions. IoU is defined as:

(9) $$IoU=\frac{S_{\mathrm{intersection}}}{S_{\mathrm{union}}},$$

where $S_{\mathrm{intersection}}$ is the shared area between the detected regions and the ground-truth regions, and $S_{\mathrm{union}}$ is the total area of the two. Higher IoU indicates tighter enclosure.

### 3.4. Experimental Results

In this section, we conducted a series of experiments to evaluate the proposed MOSI-LPD. The same experiments were also performed on corresponding baseline models for comparison. In the first experiment, we randomly sampled 10,000 images containing license plates with different orientations and multiple scales. Our MOSI-LPD was tested on the test subset for a brief overview on its performance. In the second experiment, we constructed three test subsets based on the skew degrees of license plates. These subsets were applied to evaluate the proposed strategy for multi-oriented detection based on bounding parallelograms. In the third experiment, we built three test subsets according to the scales of license plates. These subsets were used to evaluate the proposed strategy for scale-invariant detection. In the fourth experiment, we measured robustness of our MOSI-LPD to challenging data with blurs and noises. In the last experiment, we evaluated the detection speed of our MOSI-LPD. Implementation details and experimental results are presented and discussed in the following.

#### 3.4.1. Overall Performance

We randomly sampled 10,000 images from all test data to construct the test subset “Dataset10000” for this experiment. License plates in “Dataset10000” had different orientations and multiple scales. We tested the proposed MOSI-LPD on this subset.

**Figure 4:**
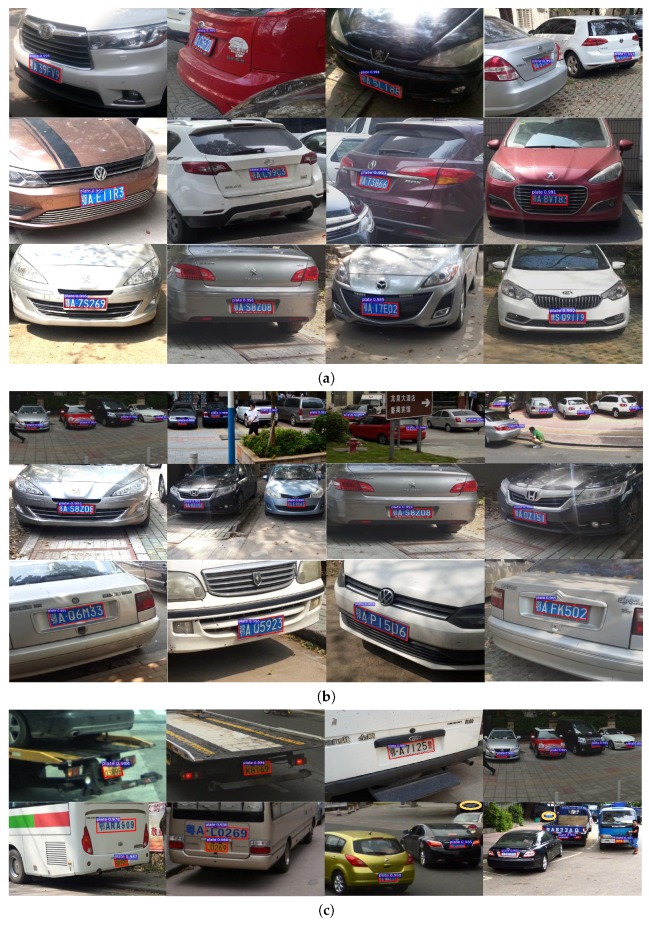
Some representative detection results of our **MOSI-LPD**: (**a**) results on license plates with different orientations (skewing violently, modestly and slightly for each row); (**b**) results on license plates with multiple scales (tiny, medium, and large in scale for each row); (**c**) results on special or low-resolution license plates in the first row, and scarce cases of mistaking or missing of license plates (indicated by yellow ellipses) in the second row.

Figure 4 shows some representative detection results. From visual inspection, we find that our MOSI-LPD effectively detects license plates with different orientations (Figure 4a) and multiple scales (Figure 4b). More importantly, the bounding parallelograms tightly enclose the multi-oriented license plates. Besides, our MOSI-LPD also successfully detects and tightly encloses special or low-resolution license plates, which are exemplified in the first row of Figure 4c. Nevertheless, there still exist very few cases in which we mistake other objects for license plates or miss the desired targets. As shown in the second row of Figure 4c: the mistakes are mostly caused by disturbance of objects similar to license plates; the miss of targets is mainly caused by the very limited information, since the license plates are so vague in the images that even human eyes can hardly figure them out.

For in-depth statistical analysis, we calculate precision, recall, f-measure and average IoU (cf. Section 3.3) of the detection results. The performance of our **MOSI-LPD** is compared with those of state-of-the-art LPD methods:

- Traditional method based on boundary features and color features [22], which is denoted as **BOCO-LPD**;
- Backbone region-based deep learning method of **Faster R-CNN** [27].

Table 1 reports the statistical analysis of the overall performance. We can find that our **MOSI-LPD** significantly outperforms **BOCO-LPD** and **Faster R-CNN** on all the evaluation metrics. Considering the enclosure performance, the average overlap between detection results and ground-truth regions achieved by our **MOSI-LPD** is about 89%, which is around 15% higher than **BOCO-LPD** and **Faster R-CNN**. This proves that our **MOSI-LPD** encloses the multi-oriented license plates in a much tighter way. Besides, considering the recognition performance, our **MOSI-LPD** obtains the highest precision and recall among these methods with the f-measure as high as 0.98. This proves that our **MOSI-LPD** achieves state-of-the-art detection performance in LPD.

Overall, the experimental results prove that our MOSI-LPD tightly encloses the multi-oriented license plates with bounding parallelograms, and is highly invariant to the scale discrepancy of license plates.

#### 3.4.2. Multi-Oriented Detection Based on Bounding Parallelograms

In this section, we evaluated our strategy for multi-oriented detection which aimed at tightly enclosing license plates with bounding parallelograms (cf. Section 2.2). The proposed strategy, namely **MO-LPD**, is compared with the original **Faster R-CNN** [27], and state-of-the-art multi-oriented text detection algorithms of **RRPN** [35] and **TextBoxes++** [37]. For this experiment, we constructed three test subsets based on skew degrees of the license plates: “Slight”, “Modest” and “Severe”. Each of the subsets was comprised of 3500 images. The first “Slight” subset contained license plates that were skewed within 5 degrees. The second “Modest” subset had license plates skewed between 5 and 25 degrees while license plates in the last “Severe” subset were skewed over 25 degrees. We evaluated **MO-LPD**, **Faster R-CNN**, **RRPN** and **TextBoxes++** on these three test subsets with visual inspection and statistical analysis.

**Figure 5:**
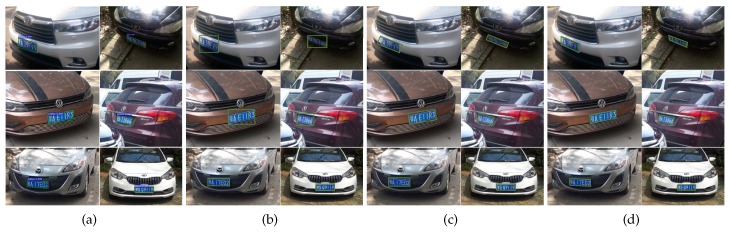
Some representative detection results: (**a**) **MO-LPD**; (**b**) **Faster R-CNN** [27]; (**c**) **RRPN** [35]; (**d**) **TextBoxes++** [37]. In each subfigure, license plates in the first to the last row were severely, modestly, and slightly skewed, respectively. The parallelograms predicted by **MO-LPD** contain less redundant information than the horizontal rectangles predicted by **Faster R-CNN** and rotated rectangles predicted by **RRPN**.

For visual inspection, Figure 5 shows some representative detection results. We find that **MO-LPD** tightly encloses the multi-oriented license plates with bounding parallelograms. As the skew degree of license plates increases, the horizontal rectangles predicted by **Faster R-CNN** contain much redundant information (shown in Figure 5b), and the rotated rectangles predicted by **RRPN** fail to enclose the corners of the license plates well (shown in Figure 5c). In contrast, the parallelograms predicted by **MO-LPD** still tightly enclose the multi-oriented license plates (shown in Figure 5a). Please note that **TextBoxes++** can also predict precise locations with quadrilaterals (shown in Figure 5d). However, according to the statistical analysis reported in Table 2, TextBoxes++ fails to guarantee the enclosure precision and detection accuracy simultaneously.

For statistical analysis, Table 2 reports the performance comparison between the methods. We find that on all of the three test subsets, **MO-LPD** obtains higher average IoU than **Faster R-CNN** and **RRPN**: the average IoU of **Faster R-CNN** is less than 80% on the “Modest” test subset, and further drops to less than 60% on the “Severe” test subset. Similarly, the average IoU of **RRPN** is less than 85% on the “Modest” test subset, and further drops to less than 75% on the “Severe” test subset. However, **MO-LPD** retains the high IoU of over 85% on all the three test subsets. For the “Severe” subset, **MO-LPD** improves the average IoU of **Faster R-CNN** and **RRPN** by around 25% and 15%, respectively. The tighter enclosure is attributed to the fact that the structures of parallelograms are more flexible than those of rectangles. As shown in Figure 5, the orientations of horizontal rectangles are fixed as 0 degree, while license plates in natural scene images are multi-oriented with various rotation degrees. The inconsistency in orientations brings redundant regions into the skewed locations. Although rotated rectangles can be freely oriented, their angles are fixed as 90 degrees. The restricted angle still results in deviations around the corners. By contrast, we regress relative positions of edge points to form bounding parallelograms. Without any restriction on either the orientation or the angle, the bounding parallelograms fit the multi-oriented regions more flexibly, and the superiority is most significant on the severely skewed license plates.

Please note that the IoU of **MO-LPD** is slightly lower than that of **TextBoxes++**, while the f-measure is much higher. The slight under-performance in IoU is attributed to the fact that the shapes of parallelograms are less free than those of quadrilaterals. As shown in Figure 5, the edge points of parallelograms should be symmetric, while the edge points of quadrilaterals can be freely located. Therefore, parallelograms are relatively less accurate than quadrilaterals in dealing with distortions. However, the inferiority is nearly negligible in most cases since the average decrease in IoU is less than 2%. By contrast, the superiority of **MO-LPD** over **TextBoxes++** in recognition performance is significant, which is indicated by an about 5% increase in f-measure. The out-performance is attributed to the less complicated task and the benefit of geometric constraints. By estimating parallelograms via predicting three edge points, the last point is easily inferred based on the symmetry property. The task becomes simpler and the prediction difficulty is reduced. In addition, the geometric constraints between diagonal points are enforced in the loss to help model training. With the robust region-based architecture, the well-trained **MO-LPD** achieves accurate enclosure precision and high detection accuracy simultaneously.

Overall, the experimental results demonstrate that **MO-LPD** encloses the multi-oriented license plates in an accurate way, and achieves high detection accuracy. The improved enclosure performance proves that our strategy for multi-oriented detection based on bounding parallelograms is effective.

#### 3.4.3. Scale-Invariant Detection

In this section, we evaluated our strategy for scale-invariant detection which aimed at detecting license plates with multiple scales (cf. Section 2.3). We compared our **MOSI-LPD** with **MO-LPD** introduced in Section 3.4.2. The difference in their performance arose from the proposed strategy for scale-invariant detection. For this experiment, we constructed three test subsets based on the scales of license plates: “Tiny”, “Medium” and “Large”. There were 3500 images in each of the subsets. The first “Tiny” subset contained license plates smaller than 300 pixels and the second “Medium” subset had license plates between 300 to 1200 pixels, while license plates in the last “Large” subset were bigger than 1200 pixels. We evaluated our **MOSI-LPD** and **MO-LPD** on these three test subsets with visual inspection and statistical analysis.

**Figure 6:**
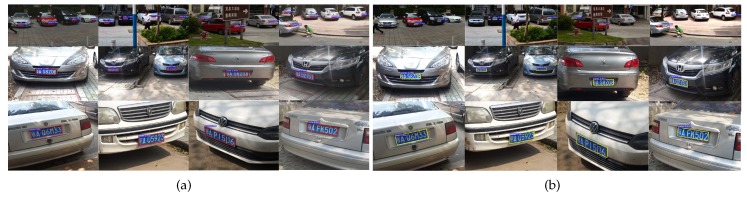
Some representative detection results: (**a**) our **MOSI-LPD**; (**b**) **MO-LPD**. In each subfigure, license plates in the first to the last row were tiny, medium, and large, respectively. Our **MOSI-LPD** is more invariant to the scale discrepancy of license plates.

For visual inspection, Figure 6 shows some representative detection results. We find that our **MOSI-LPD** manages the problem of multi-scale detection better than **MO-LPD**. As the scale of license plates decreases, **MO-LPD** fails to detect some license plates, especially the tiny ones. In contrast, our **MOSI-LPD** successfully detects license plates with multiple scales. For the tiny license plates that are hardly recognizable even by human eyes, our **MOSI-LPD** still figures them out.

For statistical analysis, Table 3 reports the performance comparison between our **MOSI-LPD** and **MO-LPD**. We find that on all the three test subsets, our **MOSI-LPD** outperforms **MO-LPD**, and the improvement is particularly significant on small license plates. Specifically, the f-measure of **MO-LPD** is only 0.93 on the “Medium” test subset, and drops to 0.9 on the “Tiny” test subset. However, the f-measure of our **MOSI-LPD** always retains higher than 0.95. On the especially challenging “Tiny” test subset, **MOSI-LPD** improves the recall and precision of **MO-LPD** by around 10%.

Overall, the experimental results demonstrate that our **MOSI-LPD** attains higher invariance to license plate scale discrepancy. The improved performance proves that our strategy to achieve multi-scale detection is effective.

#### 3.4.4. Robustness

In this section, we evaluated robustness of our **MOSI-LPD** to challenging conditions. We manually blurred and added noise to 1821 unprocessed test images. Detection performance on the disturbed images and original ones were compared and analyzed with visual inspection and statistical analysis.

**Figure 7:**
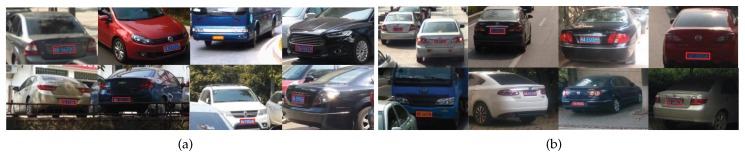
Some representative detection results of our **MOSI-LPD** on challenging data: (**a**) performance on blurred images; (**b**) performance on images with noise.

For visual inspection, Figure 7 shows some representative detection results. We can find that our **MOSI-LPD** is notably robust to tough environments. Although the license plates are quite indistinct in the disturbed images, our **MOSI-LPD** still successfully detects and tightly encloses them.

For statistical analysis, Table 4 reports the performance comparison on different conditions. We find that our **MOSI-LPD** is almost not influenced by degradation of image quality: the f-measure on the blurred data decreases by as low as around 1% and the average IoU decreases by less than 2%. In addition, on images with noises, both the f-measure and the average IoU retain as high as those on the original images.

Overall, the experimental results demonstrate that our **MOSI-LPD** is highly robust to challenging conditions. Despite the poor image quality, our **MOSI-LPD** still successfully detects and tightly encloses the license plates with bounding parallelograms.

#### 3.4.5. Detection Speed

In this section, we evaluated the detection speed of our **MOSI-LPD**. For this experiment, our **MOSI-LPD** and the backbone framework **Faster R-CNN** [27] were assessed on various test subsets: (i) “Dataset10000” (cf. Section 3.4.1); (ii) “Slight”, “Modest” and “Severe” (cf. Section 3.4.2); (iii) “Tiny”, “Medium” and “Large” (cf. Section 3.4.3). The implementation environment was introduced in Section 3.2. We recorded the average time costs of the shared fundamental convolution neural network (Conv), unshared structure of the RPN (Proposal) and unshared structure of the detection sub-network (Detection), respectively. Based on these statistics, we further reported the average detection time of the overall system.

Table 5 shows the experimental results. We find that the detection speed of our **MOSI-LPD** is remarkable. For an input image of about 1000×600 pixels, the total detection time of our **MOSI-LPD** is about 0.217s. Compared with **Faster R-CNN**, the well-known towards real-time detection network, our **MOSI-LPD** is just slightly slower. The nearly negligible decrease results from the modifications on the network architecture. However, since the extra time cost is as little as 0.041s, it almost does not affect the overall detection speed.

Overall, the experimental results demonstrate that our **MOSI-LPD** almost retains the high detection speed of the original **Faster R-CNN**, and is very fast and promising for practical LPD tasks.

## 4. Conclusions

In this paper, we proposed a MOSI-LPD based on CNNs. The proposed MOSI-LPD tightly encloses license plates with bounding parallelograms, which is much more accurate than traditional methods based on horizontal rectangles. In addition, our MOSI-LPD is highly invariant to the scale discrepancy of license plates. It can even detect tiny license plates that are only several pixels. Besides, our MOSI-LPD is robust to challenging conditions with a comparable detection speed.

## Figures and Tables

**Table 1:** Performance comparison between **BOCO-LPD** [22], **Faster R-CNN** [27] and our **MOSI-LPD** on overall performance.

Method	IoU	Precision	Recall	F-Measure
**BOCO-LPD** [22]	0.72	0.86	0.84	0.85
**Faster R-CNN** [27]	0.76	0.92	0.88	0.90
**MOSI-LPD** (ours)	**0.89**	**0.98**	**0.98**	**0.98**

**Table 2:** Performance comparison between **MO-LPD**, **Faster R-CNN** [27], **RRPN** [35] and **TextBoxes++** [37] on the “Slight”, “Modest” and “Severe” test subsets.

	Method	MO-LPD				Faster R-CNN [27]				RRPN [35]				TextBoxes++ [37]			
Dataset		IoU	Precision	Recall	F-Measure	IoU	Precision	Recall	F-Measure	IoU	Precision	Recall	F-Measure	IoU	Precision	Recall	F-Measure
Slight		0.93	0.94	0.95	**0.94**	0.90	0.94	0.93	0.93	0.91	0.91	0.95	0.93	**0.93**	0.93	0.91	0.92
Modest		0.89	0.92	0.92	**0.92**	0.78	0.87	0.92	0.90	0.83	0.93	0.91	**0.92**	**0.91**	0.84	0.87	0.85
Severe		0.87	0.89	0.87	**0.88**	0.59	0.91	0.86	**0.88**	0.72	0.88	0.84	0.86	**0.89**	0.83	0.83	0.83

**Table 3:** Performance comparison between our **MOSI-LPD** and **MO-LPD** on the “Tiny”, “Medium” and “Large” test subsets.

	Method	MOSI-LPD (ours)				MO-LPD			
Dataset		Precision	Recall	F-Measure	IoU	Precision	Recall	F-Measure	IoU
Tiny		0.98	0.96	**0.97**	0.92	0.87	0.86	0.86	0.91
Medium		0.99	0.98	**0.98**	0.91	0.96	0.91	0.93	0.88
Large		0.95	0.99	**0.97**	0.88	0.83	0.98	0.90	0.85

**Table 4:** Comparison between performance on original data and challenging data.

Testdata	Precision	Recall	F-Measure	IoU
Original Images	0.99	0.98	**0.98**	**0.93**
Blurred Images	0.97	0.97	0.97	0.91
Images with Noises	0.97	0.99	**0.98**	**0.93**

**Table 5:** The average time costs of our **MOSI-LPD** and backbone framework **Faster R-CNN** [27] on various test subsets (unit: second).

Test Subset	MOSI-LPD (ours)				Faster R-CNN [27]			
	Conv	Proposal	Detection	Total	Conv	Proposal	Detection	Total
Dataset10000	0.133	0.022	0.061	0.216	0.126	0.009	0.043	0.178
Slight	0.143	0.018	0.059	0.220	0.132	0.014	0.040	0.186
Modest	0.126	0.013	0.074	0.213	0.121	0.009	0.039	0.169
Severe	0.136	0.016	0.068	0.220	0.123	0.012	0.036	0.171
Tiny	0.137	0.014	0.057	0.208	0.127	0.007	0.041	0.175
Medium	0.142	0.018	0.063	0.223	0.119	0.011	0.038	0.168
Large	0.128	0.024	0.065	0.217	0.134	0.016	0.034	0.184
